# Achievement of higher biomass, yield and quality of essential oil of *Tagetes minuta* L. through optimizing the sowing method and seeding rate

**DOI:** 10.3389/fpls.2023.1133370

**Published:** 2023-06-08

**Authors:** Probir Kumar Pal, Mitali Mahajan, Babit Kumar Thakur, Priya Kapoor, Shivani

**Affiliations:** ^1^ Division of Agrotechnology, Council of Scientific and Industrial Research-Institute of Himalayan Bioresource Technology (CSIR-IHBT), Palampur, India; ^2^ Academy of Scientific and Innovative Research (AcSIR), Ghaziabad, India, 201002

**Keywords:** sowing method, seeding rate, broadcasting, essential oil, Z-β-ocimene, dihydrotagetone

## Abstract

*Tagetes minuta* L. is known as an industrial crop in the world as it possesses an essential oil that is extensively used in the perfumery and flavor industries. The crop performance is influenced by the planting/sowing method (SM) and seeding rate (SR); however, the effects of these variables on biomass yield and quality of the essential oil of *T. minuta* remain unclear. As a comparatively new crop, the responses of *T. minuta* to different SMs and SRs have not been studied in the mild temperate eco-region. Thus, the biomass and essential oil yield response of *T. minuta* (variety ‘Himgold’) to SM (line sowing and broadcasting) and SR (at 2, 3, 4, 5, and 6 kg ha^−1^) were investigated. The overall fresh biomass of *T. minuta* ranged from 16.86 to 28.13 Mg ha^−1^, while the essential oil concentration in fresh biomass varied from 0.23% to 0.33%. Irrespective of the SR, the broadcasting method produced significantly (*p* ≤ 0.05) higher fresh biomass yield by approximately 15.8% and 7.6% compared with line sowing during 2016 and 2017, respectively. An increase in biomass yield was noted as the SR increased up to 4 kg ha^−1^. The SR at 4 kg ha^−1^ registered approximately 41.9%–56.1% and 3.3%−10.3% higher biomass yield than the SR at 2 and 6 kg ha^−1^, respectively. No significant (*p* ≥ 0.05) differences in essential oil concentration in fresh biomass were observed due to the different SMs and SRs. Thus, *T. minuta* may be sown by the broadcasting method in the mild temperate eco-region with an SR of 4 kg ha^−1^.

## 1 Introduction

*Tagetes minuta* L. is an annual, branched, and essential oil-bearing herb. It belongs to the family of Asteraceae and is commonly known as wild marigold in India. This species has originated from the temperate grasslands of South America and has been consciously distributed across the tropics, subtropics, and several temperate countries for different purposes (Holm et al., 1997). However, *T. minuta* is mostly grown for its essential oil that is present in the macroscopic punctate oil glands in the aboveground fresh biomass. *Tagetes* essential oil is generally characterized by a higher percentage of dihydrotagetone, E- and Z-tagetone, E-ocimenone, Z-ocimenone, Z-β-ocimene, limonene, and α-terpineol.

The essential oil of *T. minuta* has high demand in the global market. Globally, the annual production of essential oil of *T. minuta* was approximately 15 tons in 2016 with a market price of $50 per kg (Cornelius and Wycliffe, 2016). The demand for essential oil of *T. minuta* is constantly increasing at a compound annual growth rate (CAGR) of 6.5% from 2020 to 2025 (by IndustryARC). The agrochemical, perfumery, pharmaceutical, food, flavor, and cosmetic industries are the major users of *T. minuta* essential oil. Thus, *T. minuta* has been cultivated in South Africa, Argentina, Australia, Brazil, Bolivia, Chile, France, Hawaii, India, Madagascar, Nigeria, Uruguay, Kenya, and the Chaco region of Paraguay (Soule, 1994; Singh et al., 2003). In India, *T. minuta* is naturally distributed in the western Himalayan regions between the altitudes of 1,000 and 2,500 masl (Thappa et al., 1993). Thus, it can be commercially grown in mild temperate to temperate regions with appropriate agronomic practices. Increased market demand and adoption of *T. minuta* in hilly temperate and mild temperate regions have provided opportunities to make the transition from subsistence farming to commercial farming and diversification of crops. The sowing method (SM) and seeding rate (SR) are both important agronomic factors involved in producing a high yield. Since *T. minuta* is a relatively new crop, there is a pressing need to evaluate the role of SM and SR to improve yield and quality.

In India, *T. minuta* seeds are sown by both broadcasting and line sowing methods. The productivity of a crop is largely governed by population density and even distribution (Olsen et al., 2005; Boyd et al., 2009). In the case of *T. minuta*, it is very difficult to maintain optimum population density and even distribution for both the sowing methods since the size of seeds is very small. The right sowing methods and seeding rates help small seeds become established rapidly and successfully. There is no standardized SM and SR for commercial cultivation. Planting/SM has significant effects on resource utilization like water, nutrient, space, and soil compaction (Troedson et al., 1989). Moreover, the absorption of photosynthetically active radiations has also been found to be influenced by planting methods (Lal et al., 1991). The correct sowing methods also ensure proper crop establishment and optimum plant population in the field and also facilitate plants to utilize the resources more efficiently toward growth and development (Gul et al., 2015). Choosing the correct SM is a basic rule for the realization of the full genetic potential of a variety, which can be achieved by implementing proper crop geometry. It affects better light and CO_2_ utilization by plant canopies, plants per unit area, and the microenvironment in and around plant canopies. Therefore, studies are required to determine the suitability of different sowing methods on a site- and crop-specific basis. An effective SM also improves the physical properties of the soil and enhances the germination rate, plant growth, and ultimately yield (Krause et al., 2009). Similarly, planting uniformity also increases crop competitive ability (Boyd et al., 2009).

The seeding rate is another major factor that could play a significant role in improving crop growth, development, and yield (Ketema, 1993; Isidro-Sánchez et al., 2017). The seeding rate depends upon agroecological conditions, soil type, time of sowing, and other agronomic factors (Isidro-Sánchez et al., 2017). Seeding rate influences interplant competition (Park et al., 2003), pathogens, soil moisture, and N availability (Fischer et al., 1976; Read and Warder, 1982). On the other hand, beyond the optimal SR or population causes a decline in yield due to intraspecific competition (Holliday, 1960; Park et al., 2003; Mahajan et al., 2021) or lodging (Berry and Spink, 2012). However, the optimal plant population/density or SR is attuned based on the availability of natural resources (Arduini et al., 2006), soil properties (Gan et al., 2009), climatic conditions (Holliday, 1960; Anderson et al., 2004), variety (Kirkegaard and Hunt, 2010), and time of plantation or sowing (Lindsey and Thomison, 2016).

The yield variations in response to different SR/planting densities have been reported in many crops like *Eragrostis tef* (Zucc.) Trotter (Mengie et al., 2021), *Triticum aestivum* L. (Twizerimana et al., 2020), *Secale cereale* L. (Boyd et al., 2009), *Triticum turgidum* L. var. *durum* (Isidro-Sánchez et al., 2017), *Pisum sativum* (Stepanovic et al., 2018), and *Zea mays* (Assefa et al., 2016). However, such type of information is not available for *T. minuta.* At the field level, the biomass and essential oil yields of *T. minuta* under different SMs and SRs have received intermittent and partial attention in the earlier scientific literature. Information is mainly available as institute extension reports (IHBT and Himgold, 2001). The effects of plant nutrition, planting date, and organic manure on biomass and essential oil yields have been tested with different crop geometries like plant-to-plant and row-to-row distances of 45 cm (Ramesh and Singh, 2008; Pandey et al., 2015) and 60 cm row spacing for line sowing (Walia and Kumar, 2021). The population density (no. of plants per unit area) has not been specifically mentioned in these reports. Nevertheless, there are no earlier reports on optimal SR and method of sowing for *T. minuta* for mild temperate conditions. Moreover, to our knowledge, previous studies have not compared manual broadcasting *vs*. manual line sowing methods for establishing a uniform and sufficiently dense higher biomass yield as well as essential oil yield of *T. minuta*. Marginal farmers basically used manual sowing methods. In the farmers’ fields, direct sowing methods either by broadcasting or line sowing are generally used without a recommended dose of SR. The scope for improving biomass yield as well as essential oil yield of *T. minuta* through suitable SM and SR combinations has not yet been fully exploited. Accordingly, we hypothesized that individual and combined agronomic factors of SM and SR could influence the establishment of the plant, biomass yield, essential oil yield, and composition of essential oil of *T. minuta* in mild temperate conditions. Therefore, to address this information gap, the primary objectives of this study were 1) to find out the suitable sowing methods for higher productivity, 2) to determine the optimal SR for the cultivation of *T. minuta* in the mild temperate region, and 3) to understand the profiling of essential oil of *T. minuta* in response to different SMs and SRs.

## 2 Material and methods

### 2.1 Experimental site, agroecology, and soil properties

**Figure 1:**
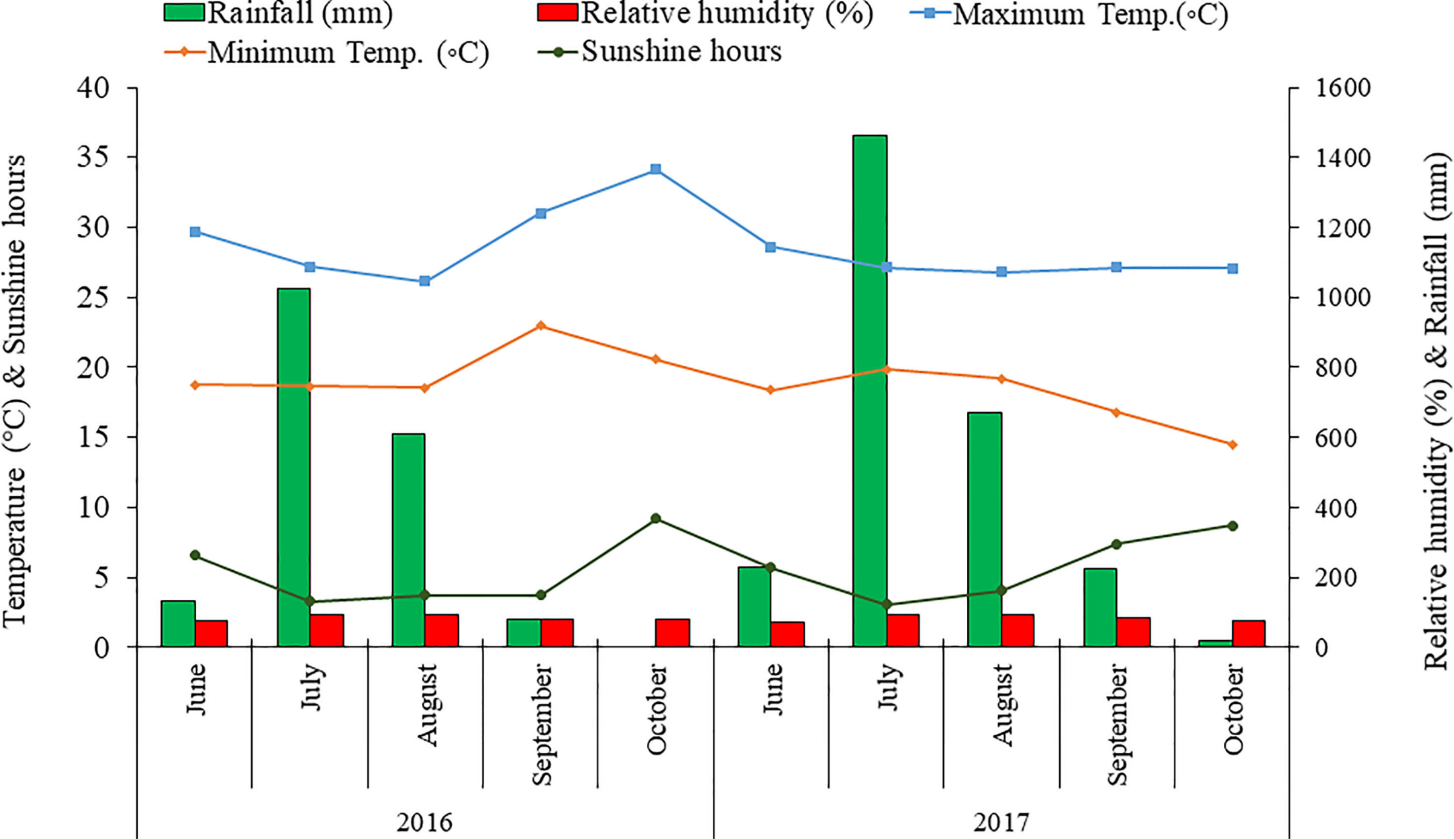
Monthly mean maximum and minimum temperature (°C), rainfall (mm), sunshine hours, and relative humidity (%) during the growing season of 2016 and 2017 at CSIR-IHBT, Palampur (HP), India.

This study was carried out at the experimental farm of CSIR-Institute of Himalayan Bioresource Technology, Palampur (32°06′05″N; 76°34′10″E,1393 m altitude) India, during the cropping seasons of 2016 and 2017. The mean annual rainfall of the experimental region is approximately 250 cm, and the mean annual temperature is 18°C. The weather parameters, viz., maximum and minimum temperature, relative humidity, and rainfall, during the crop seasons are shown in **Figure 1**. As per the USDA soil taxonomy classification system, the soil of the experimental region (Palampur) belongs to Alfisols (Sharma and Kumar, 2003). The physicochemical properties of the initial soil samples (0-15 cm depth) were studied before experimenting. The soil type of the experimental plots is silty clay, acidic in reaction (pH 5.52) having high organic carbon (1.49%) and medium available N (285.38 kg ha^−1^), available P (14.56 kg ha^−1^), and available K (256.59 kg ha^−1^).

### 2.2 Plant material, crop management, and application of treatments

#### 2.2.1 Plant material

Before the experiment, the land was fallowed for 7 months. The experiment was laid out in a two-factor factorial arrangement in a randomized block design (RBD) with three replications. Before the final layout and seed sowing, the field was properly plowed two times at 15-day intervals. Each plot has a width of 3 m and a length of 5 m. In this experiment, a total of 10 treatment combinations involving two types of SM (line sowing and broadcasting) and five levels of SR (seed rate at 2, 3, 4, 5, and 6 kg ha^−1^) were tested to standardize the SM and SR for attaining higher yield and quality of essential oil of *T. minuta* in the western Himalayan mild temperate conditions. The seeds were obtained from our own department (Agrotechnology Division, CSIR-IHBT, Palampur). The ‘Himgold (IHBT. MARIGOLD. I)’ variety was used for this study. This variety is commercially available in India. The average aboveground biomass yield of ‘Himgold’ is 20 to 25 Mg ha^−1^, and the oil concentration in fresh biomass is approximately 0.30% (IHBT and Himgold, 2001). Another important feature of this variety is synchronous flowering. The weight of 1,000 seeds ranges from 458 to 472 mg (average 465 mg). The germination percentage of seeds at the start of the experiment was approximately 87% at laboratory conditions (temperature: 25°C ± 2°C; relative humidity: 85% ± 5%).

#### 2.2.2 Application of treatments

In the case of the line sowing, the row-to-row distance was 60 cm, and the seeds were sown at the beginning of June in both years. The result of the experiment on the effect of different row-to-row distances on the yield and quality of *T. minuta* is not available. However, different authors have used different crop geometries like plant-to-plant and row-to-row distances of 45 cm (Ramesh and Singh, 2008; Pandey et al., 2015) and 60 cm row spacing for line sowing (Walia and Kumar, 2021) in other experiments. Thus, based on growth habits like height and horizontal spreading nature, the row-to-row distance of 60 cm has been used in this experiment. The sowing depth for line sowing was approximately 1-2 cm. In the case of the line sowing, the number of rows in each plot was 6. For broadcast sowing, seeds were mixed with approximately 1 kg of fine soil for uniform spreading in the experimental unit.

#### 2.2.3 Crop management

After manual broadcasting, a manual combing operation was done by a secondary tillage implement to cover the seeds. Uniform doses of N, P_2_O_5_, and K_2_O were used at 80, 40, and 80 kg ha^−1^, respectively, in both the cropping seasons. A half rate of N and full rates of P_2_O_5_ and K_2_O were applied at planting, and the remaining N was applied during the active vegetative stage. The three manual weeding practices were conducted at 20-day intervals for the initial 60 days; thereafter, weeding operations were not required. The sowing date, fertility, range of seeding rate, etc. were decided based on the information available in the institute extension reports (IHBT and Himgold, 2001) and other research reports (Ramesh and Singh, 2008; Pandey et al., 2015). Similarly, irrigation operation was done as per the requirement (3 days of intervals up to an initial 15 days) for better germination and growth of *T. minuta*, and the plots were irrigated to field capacity.

### 2.3 Agronomic trait

The agronomic traits recorded for all plots were plant height (cm), number of primary branches per plant (no. plant^−1^), and fresh biomass yield (t ha^−1^). The plant number in a unit area (no. m^−2^) has also been recorded. The crop was harvested at the end of September when all plots reached the full flowering stage. The full flowering stage is the indicator for harvesting (IHBT and Himgold, 2001). The whole plants (upper ground parts) are used for the extraction of essential oil. At harvest, the 1-m^2^ area from the center of each plot was used for recording the agronomic traits and extraction of essential oil. Plant height was recorded by measuring the distance between the base of the stem and the top of the inflorescence from the five randomly selected plants from 1.0 m^2^ quadrats. Likewise, the number of branches (no. plant^−1^) was also recorded from the same plants. The minor variability of the population within the plots was observed in both the SM. In the case of broadcasting SM, fresh biomass yield was estimated by harvesting a 1.0-m^2^ quadrat in each plot from three places. The area of 1 m from the plot boundaries was not used for sampling. In the case of the line sowing, the yield was estimated by harvesting three lines of 166.66 cm in length. Each line of 166.66 cm represents a 166.66 × 60 cm quadrat (1.0 m^2^ quadrat). Finally, the fresh biomass yield (t ha^−1^) was estimated from these 1.0 m^2^ quadrats. For the extraction of essential oil and estimation of oil concentration in fresh biomass, 1-kg subsamples were taken from each plot.

### 2.4 Extraction of essential oil

The essential oil was extracted from the fresh aerial parts of the plants harvested from each plot. The fresh samples were placed in a 5-L volumetric flask with water in a 1:2 (w/v) ratio and then hydro-distilled in a Clevenger-type apparatus for 4 h. For essential oil extraction, 1 kg of fresh aerial biomass was used. After the completion of distillation, the quantity of essential oil was recorded for the further calculation of oil concentration in the fresh aerial biomass. The extracted oil was hydrated with anhydrous sodium sulfate and then stored in sealed glass vials at 4°C in a dark place for further analysis.

### 2.5 Gas chromatography and gas chromatography-mass spectrometry analysis for the identification and quantification of volatile compounds

The essential oil extracted from all the treatments was further analyzed on a Shimadzu GC-2010 gas chromatograph (Shimadzu, Tokyo, Japan). The instrument was equipped with a flame ionization detector (FID). A DB-5 fused silica capillary column (30 m × 0.25 mm, film thickness 0.25 µm) was used with nitrogen as a carrier gas with a velocity of 1.05 ml min^−1^. The oven temperature was programmed to hold at 70°C for 4 min and to increase to 220°C at the rate of 4°C min^−1^ and finally held at 220°C for 5 min. The injector and detector temperatures were set at 240°C and 250°C, respectively. The quantification of the compounds was done based on the peak area normalization, and for each component, the response factor was considered equal to 1. Gas chromatography-mass spectrometry (GC-MS) analyses of all extracted oil samples were carried out by a Shimadzu QP2010 GC-MS system (Shimadzu, Tokyo, Japan), which was equipped with an autoinjector AOC-5000 and a DB-5 capillary column (30 m × 0.25 mm i.d. fused silica and film thickness 0.25 µm). Here, the carrier gas was helium, the flow rate was 1.1 ml min^−1^, and the other operating conditions were the same as described in Section 2.5. For compound identification, the retention indices (RIs) for all volatile compounds on the homologous series of n-alkanes (C8–C24) were calculated. The volatile components of the essential oil of *T. minuta* were identified based on a comparison of their retention indices and mass spectra with the National Institute of Standards and Technology mass spectral (NIST-MS) library (Stein, 2005).

### 2.6 Statistical analysis

**Figure 2:**
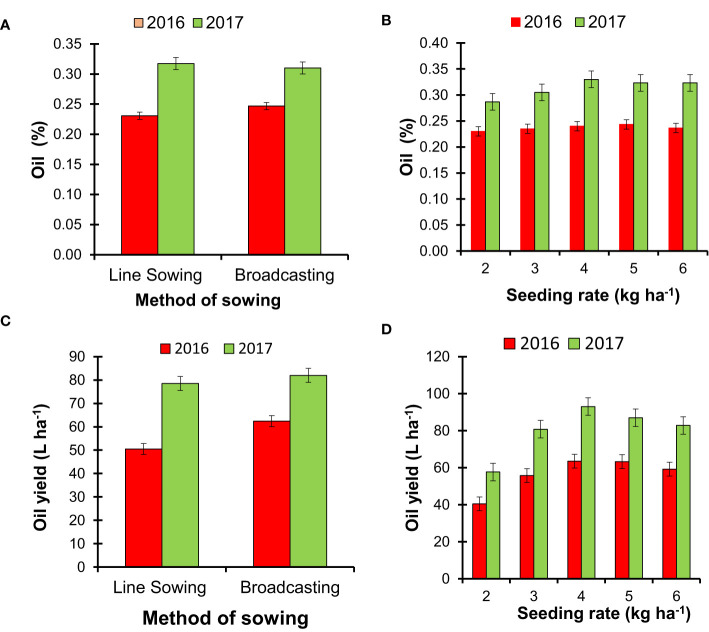
The effect of the method of sowing and seed rate of *Tagetes minuta* on the oil concentration (%) and oil yield (kg ha^−1^). The oil concentration was estimated based on the weight (w)/volume (v) basis [**(A)** method of sowing; **(B)** seeding rate], and the total oil yield (L ha^−1^) was estimated based on the total fresh biomass weight (w) and oil concentration (%) in fresh biomass [**(C)** method of sowing; **(D)** seeding rate].

The data on agronomic traits, essential oil, and compositions of essential oil were subjected to analysis of variance (ANOVA) by Statistica 7 software (Stat Soft Inc., Tulsa, OK, USA). All the analyses were conducted based on the Statistica 7 software manual. For all the traits, replications were considered random effects, whereas SM and SR were considered fixed effects during ANOVA by Statistica 7 software. To understand the biomass yield response to SR under different SMs, the regression models were also developed between the independent variable as different SRs and the dependent variable as fresh biomass yield. The correlation matrix among the agronomic traits (number of plants, plant height, number of branches, and biomass yield) and essential oil yield of *T. minuta* was also developed. Principal component analysis (PCA), a multivariate analysis technique, was conducted on the chemical profiling of essential oil to assess the influences of treatment combinations and the nature of variations among the treatment combinations by the same statistical software. This multivariate analysis explains the relationship among the variables and the nature of the influences of the treatments on the variables. The degree of the relationship between any two variables is measured by the correlation coefficient value that is calculated by the cosine of the angle between their vectors in the PCA biplot (Yan and Rajcan, 2002; Dehghani et al., 2008). Simultaneously, heat maps were prepared based on the chemical profiling of essential oil by the XLSTAT software (version 2018.2). The respective *F* values and significant level (*p-*value) obtained from Statistica 7 are also presented in **Table 1** to detect the significance of the main and interaction effects. In all parameters (**Tables 2**, **3**, **Figure 2**), the pairwise mean differences were assessed by the least significant difference (LSD) when the *F* test in ANOVA corresponding to treatments and interactions was significant at *p ≤ *0.05.

**Table 1:** F values of the analysis of variance for different parameters of *Tagetes minuta* at two sowing methods and five seeding rates grown during 2016 and 2017.

Source of variation	*df*	No. of plants (no. m^−2^)		Plant height (cm)		Branches (no. plant^−1^)		Plant yield (g plant^−1^)		Biomass yield (Mg ha^−1^)		Oil concentration (%)		Oil yield (L ha^−1^)	
		2016	2017	2016	2017	2016	2017	2016	2017	2016	2017	2016	2017	2016	2017
Sowing method (SM)	1	96.34**	35.92**	0.07	8.26**	0.08	13.74**	14.47**	13.92**	8.32**	7.85**	4.34	0.27	10.95**	0.67
Seeding rate (SR)	4	46.72**	44.56**	0.24	2.98*	1.29	6.71**	5.34**	17.97**	8.74**	19.00**	0.50	1.27	5.54**	8.13**
SM × SR	4	1.05	0.12	0.91	0.24	0.08	0.64	0.03	0.69	0.69	0.10	0.35	1.11	0.76	1.11
Source of variation	*df*	Sabinene		Limonene		Z-β-Ocimene		E-β-Ocimene		Dihydrotagetone		Alloocimene		Tagetone (E and Z)	
		2016	2017	2016	2017	2016	2017	2016	2017	2016	2017	2016	2017	2016	2017
Sowing method (SM)	1	0.07	2.28	0.18	1.07	6.38*	3.57	4.42*	0.78	1.45	2.31	4.05	0.14	17.30**	1.89
Seeding rate (SR)	4	0.75	0.50	2.17	0.29	0.86	0.86	1.26	1.06	0.34	0.93	0.62	0.74	1.84	1.04
SM × SR	4	0.16	0.61	1.01	0.68	0.14	0.62	0.55	1.08	0.19	0.66	0.29	0.15	0.76	1.08
Source of variation	*df*	Z-Ocimenone		E-Ocimenone		E-Caryophyllene		α-Humulene		Germacrene D		Bicyclogermacrene			
		2016	2017	2016	2017	2016	2017	2016	2017	2016	2017	2016	2017		
Sowing method (SM)	1	0.05	0.52	0.07	1.35	10.98**	5.42*	7.10*	4.93*	1.47	1.88	1.19	0.16		
Seeding rate (SR)	4	1.50	0.88	2.28	0.83	0.32	0.22	0.75	0.25	1.04	0.37	0.09	1.28		
SM × SR	4	1.17	1.48	1.04	0.27	0.59	0.95	1.26	1.11	0.59	0.35	0.84	1.06		

*Significant at *p* = 0.05; **significant at *p* = 0.01.

**Table 2:** Effect of the sowing method and the seeding rate on the yield attributes and yield of *Tagetes minuta*.

Treatment	No. of plants (no. m^−2^)		Plant height (cm)		Branches (no. plant^−1^)		Biomass yield (Mg ha^−1^)	
	2016	2017	2016	2017	2016	2017	2016	2017
Method of sowing (SM)								
Line sowing (L)	14.00	16.54	135.49	161.77	13.25	10.52	21.90	24.45
Broadcasting (B)	20.20	20.46	135.93	172.36	13.44	8.36	25.36	26.32
SEM (±)	0.45	0.46	1.20	2.61	0.47	0.41	0.85	0.47
CD (*p* = 0.05)	1.34	1.39	NS	7.80	NS	1.23	2.54	1.42
Seeding rate (kg ha^−1^) (SR)								
2 kg ha^−1^	9.83	10.82	135.00	155.90	14.70	12.33	16.85	19.81
3 kg ha^−1^	15.00	16.87	134.97	165.50	13.43	9.23	23.36	26.46
4 kg ha^−1^	18.33	20.07	136.93	169.57	13.33	9.03	26.31	28.13
5 kg ha^−1^	20.33	21.57	135.20	170.67	12.73	8.63	26.14	27.02
6 kg ha^−1^	22.00	23.18	136.47	173.70	12.53	7.97	25.46	25.50
SEM (±)	0.71	0.73	1.89	4.12	0.74	0.65	1.34	0.75
CD (*p* = 0.05)	2.12	2.19	NS	12.32	NS	1.95	4.01	2.24
SEM (±) (SM × SR)	0.99	1.03	2.68	5.82	1.05	0.92	1.90	1.06
CD (*p* = 0.05)	NS	NS	NS	NS	NS	NS	NS	NS

SEM, standard error of the mean; CD, critical difference; NS, not significant.

**Table 3:** Effect of the sowing method and the seeding rate on the composition of essential oil of *Tagetes minuta*.

Compounds	Year	Method of sowing				Seeding rate (kg ha^−1^)						
		Line sowing (L)	Broadcasting (B)	SEM (±)	CD (*p* = 0.05)	2 kg	3 kg	4 kg	5 kg	6 kg	SEM (±)	CD (*p* = 0.05)
Sabinene	1st	0.27	0.28	0.016	NS	0.28	0.31	0.26	0.26	0.27	0.025	NS
	2nd	0.39	0.46	0.03	NS	0.43	0.44	0.47	0.40	0.38	0.047	NS
Limonene	1st	3.86	3.76	0.167	NS	4.28	4.14	3.41	3.48	3.75	0.263	NS
	2nd	0.143	0.135	0.005	NS	0.14	0.14	0.13	0.14	0.14	0.009	NS
Z-β-Ocimene	1st	30.08	35.69	1.57	4.7	33.34	30.55	32.13	31.82	36.60	2.482	NS
	2nd	34.15	29.58	1.711	NS	29.64	31.20	29.66	33.45	35.38	2.705	NS
E-β-Ocimene	1st	0.38	0.44	0.021	0.062	0.42	0.36	0.42	0.40	0.46	0.033	NS
	2nd	0.38	0.87	0.391	NS	1.76	0.33	0.31	0.34	0.38	0.619	NS
Dihydrotagetone	1st	25.73	23.37	1.384	NS	23.98	23.51	24.46	26.74	24.07	2.189	NS
	2nd	24.14	29.04	2.284	NS	27.73	28.37	30.82	23.67	22.38	3.612	NS
Alloocimene	1st	0.70	0.82	0.044	NS	0.74	0.75	0.73	0.72	0.86	0.069	NS
	2nd	0.58	0.59	0.022	NS	0.61	0.56	0.57	0.57	0.63	0.035	NS
Tagetone (E and Z)	1st	22.26	19.05	0.545	1.63	19.94	21.79	21.86	20.52	19.17	0.862	NS
	2nd	17.16	16.14	0.445	NS	16.24	16.07	16.34	17.02	17.58	0.703	NS
Z-Ocimenone	1st	2.33	2.29	0.139	NS	2.43	2.48	2.51	2.27	1.86	0.219	NS
	2nd	3.02	2.74	0.274	NS	2.78	3.27	2.77	3.28	2.31	0.433	NS
E-Ocimenone	1st	8.24	8.10	0.365	NS	7.60	9.45	8.45	8.19	7.17	0.577	NS
	2nd	11.33	9.35	1.206	NS	11.08	8.81	8.18	11.92	11.73	1.906	NS
E-Caryophyllene	1st	0.45	0.64	0.04	0.12	0.59	0.56	0.49	0.54	0.52	0.063	NS
	2nd	0.51	0.69	0.055	0.164	0.54	0.63	0.65	0.61	0.58	0.086	NS
α-Humulene	1st	0.29	0.40	0.027	0.082	0.38	0.39	0.30	0.34	0.33	0.043	NS
	2nd	0.34	0.49	0.047	0.141	0.41	0.42	0.47	0.41	0.36	0.074	NS
Germacrene D	1st	0.10	0.13	0.014	NS	0.14	0.12	0.09	0.11	0.13	0.023	NS
	2nd	0.09	0.12	0.013	NS	0.11	0.13	0.10	0.09	0.11	0.021	NS
Bicyclogermacrene	1st	0.39	0.46	0.044	NS	0.45	0.44	0.40	0.43	0.41	0.069	NS
	2nd	0.60	0.56	0.067	NS	0.59	0.51	0.43	0.64	0.74	0.106	NS

SEM, standard error of the mean; CD, critical difference; NS, not significant.

## 3 Results

### 3.1 Yield attributes and yield

The analyzed data revealed that the number of plants per unit area (no. m^−2^) was significantly (*p* ≤ 0.05) influenced by the SM and SR during both years (**Table 2**). Irrespective of SR, broadcasting SM registered a significantly (*p* ≤ 0.05) higher number of plants per unit area (20.20 and 20.46 no. m^−2^) compared with the line SM (14.00 and 16.64 no. m^−2^) during both years. Among the SRs, the SR at 6 kg ha^−1^ produced a significantly (*p* ≤ 0.05) higher number of plants (22.00 and 23.18 no. m^−2^) compared with the other SRs in 2016 but remained statistically at par with the SR at 5 kg ha^−1^ in both years. Averaged across the SM, the SR at 6 kg ha^−1^ produced approximately 2.1–2.2 times higher number of plants per unit area compared with the SR at 2 kg ha^−1^, which produced the least numbers of plants (9.83 and 10.82 no. m^−2^) in both years. Broadcasting sowing plants registered significantly (*p* ≤ 0.05) higher plant height (172.36 cm) compared with the plants sown in line (161.77 cm) during 2017. Nevertheless, in contrast to plant height, line sowing plants produced a significantly (*p* ≤ 0.05) higher number of branches (10.52 no. plant^−1^) compared with the plants sown by broadcasting during 2017. Averaged across the SM, the SR did not alter the plant height and number of branches during the first cropping season. In the second year, the effects of SR on these parameters were significant (*p* ≤ 0.05), and the maximum height (173.70 cm) and branches (12.33 no. plant^−1^) were attained with higher SR (at 6 kg ha^−1^) and lower SR (at 2 kg ha^−1^), respectively.

**Figure 3:**
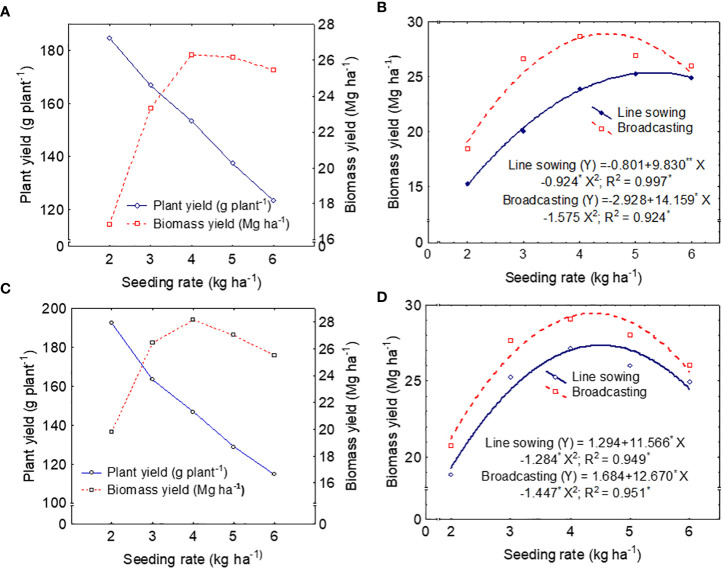
Relationship between individual fresh plant yield (g plant^−1^) and total fresh biomass yield (Mg ha^−1^) of *Tagetes minuta* at different seeding rates (SR) during 2016 **(A)** and 2017 **(B)**. Second-degree polynomial response curves represent the relationship between seeding rate (kg ha^−1^) and fresh biomass yield (Mg ha^−1^) during 2016 **(C)** and 2017 **(D)**.

Fresh biomass yield (Mg ha^−1^) of *T. minuta* was significantly (*p* ≤ 0.05) affected by the SM and SR during both years (**Table 2**), and the trend of the results was consistent over the duration of this study. Broadcasting SM produced significantly (*p* ≤ 0.05) higher biomass yield compared with line SM by approximately 15.8% and 7.6% during the first and second cropping seasons, respectively. Fresh biomass per plant decreased as SR increased up to 6 kg across the years. However, overall biomass yield (Mg ha^−1^) was increased linearly with SR up to 4 kg and thereafter declined (**Figures 3A, B**). As SR increased from 2 to 6 kg, the individual plant yields declined by approximately 33% in both years (**Figures 3A, B**). Averaged across the SM, the overall biomass yield response to SR varied significantly (*p* ≤ 0.05) as SR increased from 2 to 6 kg ha^−1^, and the utmost values (26.31 and 28.13 Mg ha^−1^) were recorded with the SR at 4 kg ha^−1^ in both years (**Figures 3A, B**; **Table 2**). As the level of SR increased from 2 to 4 kg, the total biomass yields increased by approximately 36%–56% (**Table 2**). On the other hand, the higher SR (6 kg ha^−1^) reduced biomass yield compared with the SR at 4 kg ha^−1^. The interactions between SM and SR on all the agronomic traits were insignificant (*p* ≥ 0.05) in both years.

### 3.2 Correlation and regression analysis among the agronomic traits

The correlation matrix was developed among the agronomic traits and essential oil yield to understand how yield attributes (plant density, plant height, and individual plant yield) contribute to the total biomass yield of *T. minuta* in this study (**Table 4**). The number of branches is an important yield component. However, it was not included in the correlation matrix since a weak correlation was made with biomass yield. In this experiment, significant positive correlations (*r* = 0.90, *p* ≤ 0.01 in the first year; *r* = 0.64, *p* ≤ 0.05 in the second year) were found between the number of the plant (no. m^−2^) and total biomass yield (Mg ha^−1^) in both years (**Table 4**). There were also significant but negative correlations (*r* = −0.97, *p* ≤ 0.01 in the first year; *r* = −0.91, *p* ≤ 0.05 in the second year) between individual plant yield (g plant^−1^) and the number of plants (no. m^−2^). Individual plant yield (g plant^−1^) was significantly but negatively correlated with total biomass yield (*r* = −0.81, −80; *p* ≤ 0.01) and essential oil yield (*r* = −0.82, −0.79; *p* ≤ 0.01). However, significant and positive correlations (*r* = −0.99, *p* ≤ 0.01 in the first year; *r* = −0.93, *p* ≤ 0.01 in the second year) were documented between total biomass yield and essential oil yield. Thus, mixed correlations were demonstrated in this experiment. Overall, essential oil yield increased with increasing biomass yield, and total biomass yield increased with plant density.

**Table 4:** Correlation matrix among the yield attributes, biomass yield, and essential oil yield of *Tagetes minuta*.

Parameters	No. of plants (no. m^−2^)		Plant height (cm)		Plant yield (g plant^−1^)		Biomass yield (Mg ha^−1^)		Oil yield (kg ha^−1^)	
	1st year	2nd year	1st year	2nd year	1st year	2nd year	1st year	2nd year	1st year	2nd year
No. of plants (no. m^−2^)	1.00	1.00								
Plant height (cm)	0.21	0.82**	1.00	1.00						
Plant yield (g plant^−1^)	−0.97**	−0.91**	−0.27	−0.86**	1.00	1.00				
Biomass yield (Mg ha^−1^)	0.90**	0.64*	0.31	0.77**	−0.81**	−0.80**	1.00	1.00		
Oil yield (kg ha^−1^)	0.90**	0.62	0.22	0.70*	−0.82**	−0.79**	0.99**	0.93**	1.00	1.00

*Significant at *p* = 0.05; **Significant at *p* = 0.01.

The regression models have also established between the independent variable as different SRs and the dependent variable as fresh biomass yield (**Figures 3C, D**). The quadratic model (second-degree polynomial) best explained the biomass yield–SR relationship based on the statistical model selection criterion of *R* ^2^ (*R* ^2 =^ 0.997 and 0.924 in the first year; *R* ^2 =^ 0.949 and 0.951 in the second year) values (**Figures 3C, D**). The biomass yield–SR relationship in the two SMs followed a similar trend in both years (**Figures 3C, D**) despite significant differences in actual yield levels between the two SMs. For the first year, the equations are


$$\text{Line sowing}\left(y\right)=-0.801+9.830{*}^{*}X-0.924^{*}X^{2};\left(R^{2}=0.997^{*}\right)$$

$$\text{Broadcasting}\left(y\right)=-2.928+14.159^{*}X-1.575\;X^{2};\left(R^{2}=0.924^{*}\right)$$

For the second year, the equations are


$$\text{Line sowing}\left(y\right)=1.294+11.566^{*}X-1.284^{*}X^{2};\left(R^{2}=0.949^{*}\right)$$

$$\text{Broadcasting}\left(y\right)=1.684+12.670^{*}X-1.447^{*}X^{2};\left(R^{2}=0.951^{*}\right)$$

Where *y* and *X* indicate biomass yield (Mg ha^−1^) and SR (kg ha^−1^), respectively. The biomass yield response to SR varied significantly as the SR increased from 2 to 4 kg ha^−1^ (**Table 2**, **Figures 3C, D**). During the first cropping season, the average yield increased significantly, with a slope of 4.80 Mg ha^−1^ for line sowing and 8.21 Mg ha^−1^ for broadcasting, with an SR increase from 2 to 3 kg ha^−1^ (**Figure 3C**). A moderate biomass yield increase, 3.86 Mg ha^−1^ for line sowing and 2.05 Mg ha^−1^ for broadcasting, was documented when SR was increased from 3 to 4 kg ha^−1^, but with a less relative increase, 1.35 Mg ha^−1^ for line sowing, when SR was increased from 4 to 5 kg ha^−1^. In the case of the broadcasting sowing, yield declined by 1.70 Mg ha^−1^. During the second year, a similar biomass yield increasing trend was recorded for the broadcasting SM with a slope of 6.91 and 1.43 Mg ha^−1^ when the SR was increased from 2 to 3 kg ha^−1^ and 2 to 3 kg ha^−1^, respectively (**Figure 3D**). For the higher SR, when SR was increased from 4 to 6 kg ha^−1^, yield declined by 2.21 Mg ha^−1^ for line sowing and 3.04 Mg ha^−1^ for broadcasting.

### 3.3 Oil concentration (%) in aboveground biomass and oil yield (kg ha^−1^)

The SM and SR did not alter the concentration (%) of essential oil in the aboveground fresh biomass of *T. minuta* in the present study, and the trend was consistent over the duration of this study (**Figures 2A, B**). However, higher SR registered a marginally higher concentration of essential oil (0.24% in 2016 and 0.33% in 2017) compared with low SR, and the overall concentration of essential oil in biomass was slightly higher in 2017(**Figure 2B**). The total yield of essential oil was significantly (*p* ≤ 0.05) influenced by SM in the first cropping season, and broadcasting SM produced approximately 23.4% and 4.4% higher essential oil compared with line SM during 2016 and 2017, respectively (**Figure 2C**). The effect of SR on essential oil yield was significant (*p* ≤ 0.05) in both years, and the utmost values (63.52 and 93.04 L ha^−1^) were recorded with SR of 4 kg ha^−1^ in both years (**Figure 2D**). Intermediate SR (at 4 kg ha^−1^) produced approximately 56.9%–61.3% and 7.2%–12.3% higher oil yield compared with low SR (at 2 kg ha^−1^) and high SR (at 6 kg ha^−1^), respectively.

### 3.4 Composition of essential oil

Qualitative evaluation of the essential oils of *T. minuta* under different SMs and SRs was done through the identification and quantification of the compounds (**Table 3**). In **Table 3**, we presented only 13 compounds, which were identified in all the treatments and replications. The compounds, which were not identified in all the treatments and replications, were not presented in this table. These 13 compounds contributed approximately 88.66%–96.01% of the total volume of essential oil samples obtained in this study. The data revealed that only a few compounds (E-caryophyllene, α-humulene, Z-β-ocimene, E-β-ocimene, and tagetone in the second year only) were affected by the SM in this study. Broadcasting SM produced a significantly (*p* ≤ 0.05) higher amount of E-caryophyllene and α-humulene in both years (**Table 3**). In *T. minuta* essential oil, one of the major and important compounds is Z-β-ocimene, which was significantly (*p* ≤ 0.05) influenced by the SM in the first cropping season, and maximum Z-β-ocimene content (35.69%) was recorded with broadcasting SM. However, the trend was not consistent between the years (**Table 3**). In the case of E-β-ocimene, the trend over the years was similar, and a significantly (*p* ≤ 0.05) higher value (35.69%) was recorded with broadcasting SM during 2017. The SR did not influence the composition of essential oil in this study; however, the maximum (36.60% and 35.38%) values of Z-β-ocimene were documented with the application of higher SR (at 6 kg ha^−1^) (**Table 3**).

**Figure 4:**
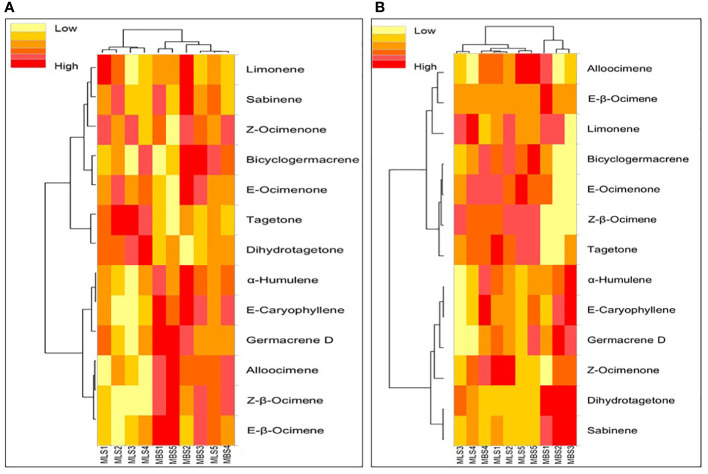
The heat map illustrating the changing pattern of the identified compounds as affected by the interaction between the sowing method (SM) and seeding rate (SR) in 2016 **(A)** and 2017 **(B)**. M_L_ and M_B_ represent the line SM and broadcasting SM, respectively, while S_1_, S_2_, S_3_, S_4_, and S_5_ are the SR at 2, 3, 4, 5, and 6 kg ha^−1^, respectively.

Variations in the chemical profile of the essential oil under the interaction effects between SM and SR have also been illustrated by heat maps (**Figures 4A, B**). A total of 13 compounds were used in the heat maps, which depict the changes in the chemical profiling of the essential oil of *T. minuta* due to the interaction between the SM and SR. Clustering was done for the chemical profile of essential oil as well as for treatment combinations by developing dendrograms (**Figures 4A, B**). The heat map exhibited that the accumulation patterns of most of the compounds were uneven over the years (**Figures 4A, B**). During the 2016 season, maximum (38.54%) and minimum (27.92%) Z-β-ocimene concentrations were recorded with M_B_S_5_ (broadcasting SM with SR at 6 kg ha^−1^) and M_L_S_4_ (line SM with SR at 5 kg ha^−1^), respectively. However, in 2017, M_L_S_5_ (line SM with SR at 6 kg ha^−1^) and M_B_S_3_ (broadcasting SM with SR at 4 kg ha^−1^) registered maximum (35.50%) and minimum (25.22%) quantities of Z-β-ocimene, respectively (**Figures 4A, B**). Dihydrotagetone is another major compound that also shows inconsistent accumulation patterns under different treatment combinations over the years. In 2016, the maximum value (29.14%) was recorded with M_L_S_4_ (line SM with SR at 5 kg ha^−1^), whereas in 2017, the maximum value (33.81) was recorded with M_B_S_2_ (broadcasting SM with SR at 3 kg ha^−1^). Moreover, the heat map also indicated that treatment combinations were divided into two major clusters in both years (**Figures 4A, B**).

### 3.5 Principal component analysis

**Figure 5:**
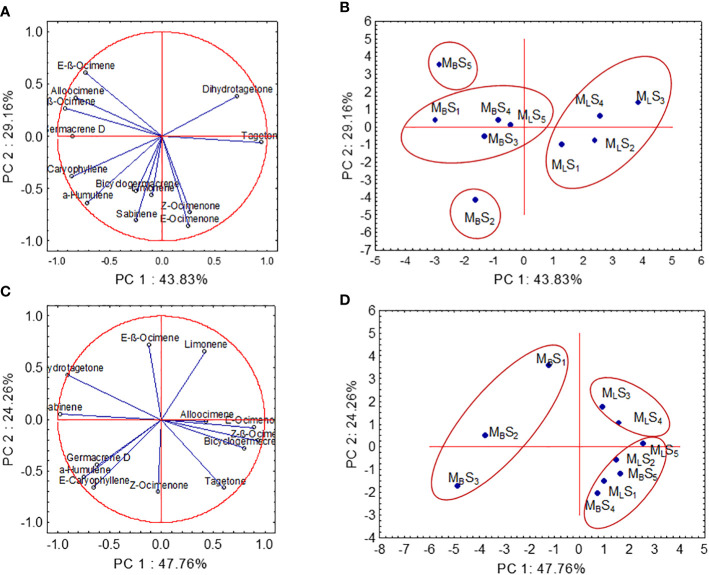
Principal component analysis of compounds of essential oil from 10 treatment combinations for both years [**(A, B)** for 2016; **(C, D)** for 2017]. Letters and numbers refer to treatment combinations: M_L_S_1_ (line sowing with seeding rate at 2 kg ha^−1^), M_L_S_2_ (line sowing with seeding rate at 3 kg ha^−1^), M_L_S_3_ (line sowing with seeding rate at 4 kg ha^−1^), M_L_S_4_ (line sowing with seeding rate at 5 kg ha^−1^), M_L_S_5_ (line sowing with seeding rate at 6 kg ha^−1^), M_B_S_1_ (broadcasting sowing with seeding rate at 2 kg ha^−1^), M_B_S_2_ (broadcasting sowing with seeding rate at 3 kg ha^−1^), M_B_S_3_ (broadcasting sowing with seeding rate at 4 kg ha^−1^), M_B_S_4_ (broadcasting sowing with seeding rate at 5 kg ha^−1^), and M_B_S_5_ (broadcasting sowing with seeding rate at 6 kg ha^−1^).

PCA analyses were performed taking into account the identified compounds in essential oil under different SMs and SRs for both years (**Figures 5A–D**). In this study, the PCA biplot exhibited that the first component (PC1) and the second component (PC2) jointly explained 72.99% and 72.02% of the total variations for 2016 and 2017, respectively (**Figure 5**). The PCA biplot technique confirmed the relationship among the variables (compounds) and the nature of influences of the treatment combinations on the variables. In the PCA biplot, the vectors from the origin to each marker of the compounds enable visualization of the relationships between and among the compounds (**Figures 5A, C**). The strength of the relationship between any two variables is assessed by the correlation coefficient, which is estimated by the cosine of the angle between their vectors in the PCA biplot (Yan and Rajcan, 2002; Dehghani et al., 2008). Two variables are independent, positively correlated, and negatively correlated when the angle between their vectors is 90°, less than 90°, and greater than 90°, respectively. Similarly, variables (compounds in this study) with longer and shorter vectors have strong and weak correlations with the PCs, respectively. In 2016 (**Figure 5A**), strong positive associations between dihydrotagetone and tagetone; among Z-β-ocimene, E-β-ocimene, and alloocimene; between Z-ocimenone and E-ocimenone; and between E-caryophyllene and α-humulene were observed as exhibited by the narrow acute angles between their vectors (e.g., *r* = cos 0 = +1). Also, **Figure 5A** demonstrates the negative association between dihydrotagetone and E-caryophyllene, between dihydrotagetone and α-humulene, and between E-ocimenone and E-β;-ocimene, since large obtuse angles between their vectors were observed. Similarly, in 2016, strong positive associations between E-β-ocimene and limonene, between sabinene and dihydrotagetone, between Z-ocimenone and tagetone, and between E-caryophyllene and α-humulene were observed as exhibited by the narrow acute angles between their vectors (**Figure 5C**). Furthermore, **Figure 5C** demonstrates the negative association between E-caryophyllene and E-β-ocimene, between E-ß-ocimene and tagetone, and between E-ocimenone and E-β-ocimene, since large obtuse angles between their vectors were observed. PCA analysis based on 13 volatile compounds revealed a clear separation into four and three treatment combination groups for 2016 and 2017, respectively (**Figures 5B, D**). In 2016, line SM with SR from 2 to 5 kg ha^−1^ developed a single group and was placed in the positive end of PC1 (**Figure 5B**). Moreover, broadcasting SM with SR of 3 and 6 kg ha^−1^ developed an independent group and was separated by the PC2 (**Figure 5B**). In 2017, the PCA biplot (**Figure 5B**) showed that the broadcasting SM with SR 2, 3, and 4 kg ha^−1^ came under the same group in terms of essential oil composition; however, all three treatment combinations were placed in the negative end of PC1.

## 4 Discussion

In this experiment, broadcasting SM produced 7.6% to 15.8% more biomass yield compared with line SM due to the higher number of plants per square meter without significant reduction of other yield attributes like plant height and number of branches per plant (**Table 2**). It is evident from our present study that biomass yield (Mg ha^−1^) is positively correlated with the number of plants per square meter (*r* = 0.90, *p* ≤ 0.01; *r* = 0.64, *p* ≤ 0.05). Furthermore, under broadcasting SM, the number of plants per unit area increased by 24%–44% compared with the line SM (**Table 2**). An even distribution of seeds resulted in increasing the number of plants per square meter for broadcasting SM compared with line SM. The number of plants per unit area has an impact on plant architecture and modifies growth and development patterns (Abuzar et al., 2011). In this study, the plants did not suffer from resource limitations and, consequently, produce higher fresh biomass with broadcasting SM. The reason behind the fewer number of plants (no. m^−2^) under the line SM might be poor germination/emergence of seeds due to the overlapping of the seeds and uneven depth of seed sowing. In case of sowing too deeply, seeds must emerge through a greater volume of soil which leads to decreased emergence of plants. Seeds sown much deeper usually have a decreased germination rate or decreased emergence of seedlings due to deficiency of O_2_, light, and other factors related to germination (Van Assche and Vanlerberghe, 1989; Vleeshouwers, 1997). In another study, it has been documented that broadcasting SM produces higher yield and yield-related traits of berseem (*Trifolium alexandrinum*) compared with row sowing (Jabbar et al., 2022). In the second season, a decreased number of branches per plant was observed with broadcasting SM probably due to a shortage of space for lateral growth, which resulted in increased plant height. The negative effect of LS is also observed in jute yield due to mutual shading between plants (Palit and Bhattacharyya, 1987). The utilization of resources like water, nutrient, space, and absorption of photosynthetically active radiation is essentially influenced by planting methods (Troedson et al., 1989; Lal et al., 1991). The results of our experiment suggest that at a given SR, broadcasting crops had characteristics such as greater uniformity and crop stand, and these would likely increase aboveground biomass yield. Thus, the broadcasting method may be followed since special agronomic practices like earthing-up picking/plucking are not required.

Although plants have the capability to some extent to compensate for the production of biomass for low density/population by producing more horizontal growth, seeding above or below the optimal level may lead to the overall poor performance of the crops. For the efficient utilization of above- and belowground natural resources, optimal SR is one of the main determining factors (Lloveras et al., 2004). Within the range of SRs used (2–6 kg ha^−1^) in our experiment, the overall biomass yield (Mg ha^−1^) was increased linearly with SR up to 4 kg and thereafter decreased (**Figures 3A, B**). However, the fresh biomass per plant (g plant^−1^) decreased as SR increased up to 6 kg within the duration of this study. The quadratic relationship between SR and fresh biomass yield (Mg ha^−1^) in this experiment coincides with the findings of Holliday (1960); Faris and DePauw (1980), and Abebe and Abebe (2016). Berti and Samarappuli (2018) have also observed that neither the forage yield nor the quality of alfalfa does not improve with increased SR beyond the recommended rate. This low biomass yield of *T. minuta* with a higher SR (6 kg ha^−1^) compared with medium SR (4 kg ha^−1^) is primarily due to a higher plant population per unit area. Plant population above a certain limit negatively impacts the lighting and ventilation conditions in the population structure, which ultimately decreases the utilization rate of light energy and reduces biological and economic production. From our present results, it is evident that fresh biomass per plant (g plant^−1^) decreased as SR increased up to 6 kg across the years (**Figures 3A, B**). Plants demonstrate photomorphogenic response by altering their structure to cope with various stresses (Read and Stokes, 2006). Increased plant height and decreased number of branches were also recorded with higher SR probably due to the longer internode lengths. A dense canopy receives more far-red than red radiations (Sattin et al., 1994; Xue et al., 2016), and the increased far-red/red ratio encourages internode elongation and supports apical dominance (Troyer and Rosenbrook, 1991; Rajcan and Swanton, 2001). Thus, apical dominance may cause a decrease in the numbers or shorter lengths of branches with increased SR. In the case of apical dominance, the axillary buds start outgrowth at maturity or just before the reproductive stage. In this experiment, the number of branches was not significantly changed by the SR in the first year; however, the biomass yield was reduced with a higher SR. The effects of crowding stress have also been observed in corn yield (Averbeke and Marais, 1992; Weiner, 1993). Yield varied among different SRs, with SR at 4 kg ha^−1^ producing greater harvestable fresh biomass. Moreover, the measured biomass yield in our study averaged across SM was considerably higher than in previous reports from Indian conditions of 1.26 to 18.8 Mg ha^−1^ (Ramesh and Singh, 2008; Pandey et al., 2015). Thus, our results demonstrate that a high biomass yield can be attained in a specific environment by adjusting SR. However, planting of *T. minuta* at SR above 4 kg ha^−1^ would not be beneficial to increase the biomass yield in mild temperate conditions. No significant (*p* ≤ 0.05) yield differences occurred between SR of above 3 and 4 kg ha^−1^; nonetheless, the additional output cost (202-350 USD; 1 USD equivalent to 82.39 Indian rupees on 18 October 2022) from 1 kg of seeds was substantially higher than the cost (~24.25 USD) of seeds.

In this experiment, the essential oil concentration in the fresh biomass ranged from 0.23% to 0.33% depending on the SM, SR, and cropping seasons; nevertheless, the variations due to SM and SR were statistically insignificant (**Figures 2A, B**). A recent study showed that water limitation/stress did not alter the essential oil content of *T. minuta* but changes the essential oil composition (Babaei et al., 2021). The seasonal and temperature effects on the accumulation of essential oil have been documented in earlier studies (Najem et al., 2011; Mahajan and Pal, 2020). The yield improvement of essential oil with broadcasting SM and intermediate SR was essentially due to higher biomass yields without compromising the concentration of oil in the respective treatments. The present results are in agreement with the observation of earlier researchers (Walia and Kumar, 2021) who established a positive correlation between aboveground biomass and essential oil yield of *T. minuta*. The synthesis of essential oil is costly to the plant since essential oil is a secondary metabolite. For the synthesis of a secondary metabolite, a steady array of precursors, along with enzymes and cofactors like ATP and NADPH, is required (Harborne, 1997). The positive correlations (*r* = 99 in the first year and *r* = 93 in the second year; *p* ≤ 0.01) between essential oil yield and biomass yield in this experiment indicate the existence of a trade-off between oil concentration in fresh biomass and fresh biomass yield.

Regardless of the SM and SR, ocimenones E and Z, tagetones E and Z, dihydrotagetone, and hydrocarbon ocimene were identified as the major constituents of the essential oil of *T. minuta* in this experiment. These compounds are used as base materials to synthesize new aroma molecules (Singh et al., 2003). In this study, only E-caryophyllene, α-humulene, Z-β-ocimene, E-β-ocimene, and tagetones E and Z (second year only) were significantly changed by the SM; however, the SR did not influence the chemical profile of essential oil (**Table 3**). We have also developed a PCA biplot to identify the suitable combination of SM and SR based on the composition of essential oil. In the first season, all the SMs (except M_L_M_5_: line sowing with SR of 6 kg ha^−1^) are separated by the PC1; however, in the second season, M_B_S_1_, M_B_S_2_, and M_B_S_3_ are separated by the PC1. Data from this study suggest that there is no definite pattern of accumulation of different compounds under different SMs and SRs. However, the changes in the content of essential oils and their constituents have been reported depending on variations in agroclimatic conditions, growing season, growth/harvesting stage, plant parts, cultivation practices, and origin (Graven et al., 1991; Thappa et al., 1993; Chalchat et al., 1995; Ram et al., 1998; Bansal et al., 1999; Ram et al., 1999; Singh, 2001; Singh et al., 2006). Thus, it is clear from the results of this study that the quality of the essential oil of *T. minuta* is not influenced by the SR.

## 5 Conclusions

The results from this study suggest that SM and SR altered biomass and essential oil yield of *T. minuta*, and their effects were consistent over the duration of this study. We believe that broadcasting SM is worthwhile considering the higher emergence rate, which ultimately increased aboveground fresh biomass yield by approximately 7.6%–15.8% irrespective of SR. Averaged across the SM, the overall biomass yield response to SR changed as SR increased from 2 to 6 kg ha^−1^, and the utmost values were recorded with the SR at 4 kg ha^−1^ in both seasons. However, variations in biomass yield concentrations of essential oil were not influenced by changes in SM and SR. The results from this investigation establish the hypothesis that SM and SR modulate biomass and essential oil yield of *T. minuta*. Thus, this study leads to the conclusion that broadcasting SM is suitable for higher fresh biomass yield without reducing the concentration of essential oil, and an increase in SR up to 4 kg ha^−1^ is worthwhile to increase the biomass yield of *T. minuta* in mild temperate conditions. However, additional studies are needed in order to understand the correct sowing depth and the relationship between nutrient rate, particularly nitrogen, and plant population to increase the yield and efficient utilization of resources.

## Data availability statement

The original contributions presented in the study are included in the article/supplementary material. Further inquiries can be directed to the corresponding author.

## Author contributions

PP: funding acquisition, conceptualization, methodology, statistical analysis and interpretation, and manuscript writing and editing. MM and BT: data collection, conducting the experiment, essential oil extraction, and writing—original draft. PK and S: essential oil extraction and identification of compounds. All authors contributed to the article and approved the submitted version.
